# Smoke Inhalation Lung Injury: An Update

**Published:** 2008-05-16

**Authors:** Robert H. Demling

**Affiliations:** Harvard Medical School, Burn and Trauma Center, Brigham and Women's Hospital, Boston, MA

## Abstract

**Objectives:** The purpose of this study is to present a multifaceted, definitive review of the past and current status of smoke inhalation injury. History along with current understanding of anatomical, physiology, and biologic components will be discussed. **Methods:** The literature has been reviewed from the early onset of the concept of smoke inhalation in the 1920s to our current understanding as of 2007. **Results:** The results indicate that the current pathophysiologic concept is of a disease process that leads to immediate and delayed pulmonary injury best managed by aggressive physiologic support. Management approaches for the biochemical changes have not kept up with current knowledge. The lung injury process is activated by toxins in the smoke's gas and particle components and perpetuated by a resulting lung inflammation. This inflammatory process becomes self-perpetuating through the activation of a large number of inflammatory cascades. In addition, smoke injury leads to significant systemic abnormalities injuring other organs and accentuating the burn injury process and subsequently leading to mediator-induced cellular injury leading potentially to multisystem organ failure. **Conclusions:** Smoke inhalation injury results in the anatomic finding of denuded and sometimes sloughed airways mucosa. Physiologic findings include small airways containing fibrin casts of mucosa and neutrophils. Airway hyper-reactivity results as well, leading to further decreased collapse, causing obstruction.

Smoke inhalation injury, either by itself or in the presence of a burn, is now well-recognized to result in severe lung-induced morbidity and mortality. The most common cause of death in burn centers is now respiratory failure.^1^–^3^

Overall, burn tragedies in history have markedly improved our knowledge of this injury (Table 1). Although smoke inhalation injury has been present since ancient times, it was probably the use of chemical warfare agents in World War I that first initiated the interest.

In 1915, the German army released aerosolized chlorine into the air toward the Allied troops. The response, upon breathing, was severe airway irritation with coughing, and severe exposure led to pulmonary edema and death. Filtering the gases with an activated charcoal filter removed the chlorine from the air,^4^,^5^ a method of decreasing lung damage.

Phosgene gas, a component of smoke, was purified and also used as a chemical warfare agent along with mustard gas, also leading to lung damage.^4^ Phosgene is a common component of smoke today in any fire.

The Cleveland Clinic Fire, in 1929, was caused by burning x-ray film. The cause of the many respiratory deaths was considered to be the released toxin, nitrogen dioxide, again a component of smoke in fires today.^6^

Skin burns were frequently absent and management of the lung injury became a priority for burn teams. The pulmonary pathology after smoke exposure was considered to be slough of the large- and small-airway mucosa causing obstruction to breathing. It was not until after the Coconut Grove Night Club Fire in 1942 that a surge of research in the pathophysiology of smoke inhalation developed.^7^,^8^ In this tragedy, hundreds of people were involved, many dying rapidly from apparent respiratory failure in the absence of burns. Others initially survived only to die later of what appeared to be pneumonia.^7^,^8^

Of importance was the fact that victims, not dead on arrival, appeared to be stable until 12- to 24-hours later, at which point the respiratory distress developed.^7^–^9^ Bronchial obstructions which developed, now recognized to be the result of the delayed airways mucosal slough, were caused by toxins that carried carbonaceous particles. Carbon monoxide poisoning was then well understood, so the initial use of oxygen was already established.^9^

By the mid-1940s, World War II was underway, and the risks of lung damage from closed-space fires were well recognized. Dr Oliver Cope, at the Massachusetts General Hospital, was instrumental in describing the pathology, time course, and the treatment of the severe cases of smoke inhalation injury. Respiratory assistance was recognized as a key factor in survival.^9^

The late effect on the airways and alveoli of smoke exposure was not yet appreciated, as ventilator assistance was not yet a tool used for longer-term survival. It was not until the use of blood-gas analysis in the late 1960s, that a clearer pattern of basic pathophysiology was determined along with the establishment of critical care medicine.^10^ The adult respiratory distress syndrome (ARDS) was also becoming recognized in the 1960s, and alveolar edema and collapse were recognized.^11^

It was still to be determined that smoke inhalation was mainly a large- and small-airway injury, although ARDS remained an alveolar damage process. Also, the role of bacteria in the lungs after inhalation injury became better recognized.^12^,^13^

A burst of research activity from the late 1970s and early 1980s better defined the chemical toxins found in smoke, including cyanide. Advances in the field of toxicology and the composition of aerosols provided the tools for research in the area. Identification of gas and particle phases of smoke and its components allowed for fairly accurate cause-and-effect hypotheses.^11^–^16^

The advances were followed by more active research on particle size and distribution in the lung. It was not appreciated until later that the particles carried gas phase toxins, which then deposited on the tracheal bronchial tree leading to the airways injury. The potentiating effect of a smoke injury on burn morbidity and mortality was well described in the 1980s. The mechanism of this potentiating effect still remains poorly understood.^17^–^20^

Overall, uncovering the physiologic changes in the lung evolved stepwise through fairly recent history (Table 2).^21^–^23^

Through the 1990s to the present, the research focus in this area has been to better define the biochemical and cell biologic changes, occurrence of which would explain the recognized physiologic changes. The products of airway inflammation, characteristics of smoke injury, have been of particular interest.^24^–^29^ Proinflammatory cytokines and free oxygen radicals have been demonstrated to play a significant role in both the lung and systemic response to smoke. More recently, apoptosis or programmed cell death has been found in the injured airways epithelium.^28^

The Station Nightclub Fire in Rhode Island in 2005 and the 9/11 World Trade Center tragedy in 2001 have led to a number of observations, stimulating further research.^29^,^30^

However, it is fair to state that the recently identified cell biologic and cell genetic changes have not yet altered the clinical management. The identified physiologic changes have, however, played a major role in improving management, thereby leading to a decrease in mortality and morbidity.

## COMMON COMPONENTS OF SMOKE

The composition of smoke, which leads to the lung injury, is described in this section.

### Chemicals in smoke (Figure 1)

The majority of toxicology studies on smoke injury were performed in the 1970s and 1980s with little research since that time. There are a large number of well-described components of smoke and descriptions of their effect on the pulmonary and systemic physiologic components. The sources of these components are also described (Table 3,^31^–^35^ and 4).

**Carbon monoxide** is released during combustion of any product. Carbon monoxide leads to generalized tissue hypoxia and possible death when combining with hemoglobin.^36^,^37^

**Carbon dioxide** is also released, increasing respiratory drive and respiratory efforts.

**Hydrogen cyanide** is released from a number of products including polyurethane, used to insulate furniture and mattresses, as well as the burning of wool, silk, and carpets. The cyanide binds to the cytochrome system and leads to tissue hypoxia and possible death.^38^,^39^

The combination of carbon monoxide and cyanide likely occurs in a typical closed-space fire, especially with the increased use of household synthetics.^40^

**Hydrogen chloride** is released in large amounts with the combustion of polyvinyl, a common compound used to cover furniture, floors, and upholstery. It appears that hydrochloric acid bound to particles is much more toxic than the hydrochloric acid dissolved in the gas. The hydrochloric acid causes destruction of the airway mucosa and results in acute bronchitis (Table 5).^40^,^41^

**Phosgene** is a strong pulmonary irritant. It is a colorless gas, relatively water soluble but also carried on particles. It is released with the combustion of polyvinyl chloride. Phosgene is slowly hydrolyzed to hydrochloric acid and carbon dioxide. Primary sites of injury are the small airways and alveoli.^42^

**Acrolein**, also called propenal, is a toxic compound released with burning of wood, cotton, paper, and petroleum. It is the simplest aldehyde with a 3-carbon chain. Acrolein is a very unstable compound that can bind to particles. Severe upper respiratory irritation can occur, and it is also toxic to lower airways mucosa. Sudden death in less than 10 minutes will occur with breathing concentrations of over 50 ppm.^43^,^44^

**Other aldehydes** like formaldehyde are released with combustion of a large number of synthetics and woods. These compounds are corrosive and denature tissue proteins. Formaldehyde also denatures RNA. These agents are some of the most toxic components of smoke.

**Free radicals**, especially oxygen-free radicals, are released from virtually all burning products, especially wood and rubber. These compounds are highly reactive and, therefore, toxic to tissues. Peroxidation of the outer lipid layer of the cell is one of the results, leading to cell damage and cell death. Long-acting free radicals are generated by chemical reactions in the smoke itself. Radicals that can last more than 20 minutes are also responsible for accentuating inflammation.^3^,^45^–^48^

**Ammonia** is an irritant compound released in gas phase with the combustion of many synthetic products leading to tearing, cough, increased secretions and bronchoconstriction. Ammonia also forms ammonium hydroxide, a potent alkali leading to tissue necrosis when carried by particles to lower airways.^42^–^49^

**Sulfur dioxide** is a compound released mainly through the burning of rubber products. It is very irritating to the airway mucosa and the eyes at relatively low concentrations. High concentrations will be fatal. It is oxidized to sulfurous acid and sulfuric acid. Toxicity is increased when dissolved onto soot particles. It is associated with lower-airways injury and lung edema.^49^,^50^

**Nitrogen dioxide** is the most important nitrogen oxide. NO_2_ is a gas with limited solubility in water and can be carried by particles. It is produced by the combustion of fabrics and cellulose products. The injury is mainly to lower airways and is delayed in onset up to 72 hours. Lipid solubility leads to damage to all membranes and cell death. Excessive damage to airways and alveolar epithelium is noted.^49^,^50^

**Chlorine** is an intensely irritating compound that, when dissolved in water on the mucosal surface, forms hydrochloride and hydrochlorus acid. Oxygen radicals are also released; chlorine (Cl_2_) is released with combustion of plastics and resins and is a common bleaching agent. Chlorine is believed to cause damage to tissues because of its very potent oxidizing properties. Intense bronchospasm as well as cell necrosis results.^42^,^49^

**Fire retardants** are usually polymeric products with the insertion of halogens and phosphorous. They are used in mattresses and furniture. Combustion leads to the release of cyanide, acetylene, methane, and a variety of toxins that can damage mucosal surfaces as well as have systemic effects.

**Aromatic hydrocarbons**, like benzene, seen mainly as organic polymers, become volatile unsaturated hydrocarbons on burning leading to local airway irritability and systemic toxicity.

Interestingly, the majority of the studies assessing smoke toxicity were performed in the 1970s and 1980s, and there has not been much toxicity research since then.

### Carbon monoxide and cyanide toxicity

Carbon monoxide toxicity is one of the leading causes of death in fires. Whereas oxygen is used during combustion, carbon monoxide is released, because it is a basic by-product of combustion.^16^,^41^ Carbon monoxide is rapidly transported across the alveolar membrane and preferentially binds with the hemoglobin molecule in place of oxygen. In addition, carbon monoxide shifts the hemoglobin-oxygen dissociation curve to the left, thereby impairing oxygen unloading at the tissue level. The result is a major impairment in oxygen delivery.^51^–^53^

The absorption of CO is dependent on its concentration in the smoke exposure. Table 6 shows the anticipated concentration of CO in light, moderate, and heavy smoke. With heavy smoke, the time to toxicity (CO level of 20%) is less than a 5-minute exposure.

Production of hydrocyanide, the gaseous form of cyanide, is also a well-recognized cause of morbidity and mortality, especially with burning of synthetics such as polyurethane. The combination of carbon monoxide and cyanide as a cause of death is quite common.^16^ Although cyanide can be absorbed through the gastrointestinal tract or skin, it is most dangerous when aerosolized and inhaled because of its rapid absorption through the large surface area of the lung. The hydrocyanide then binds to the cytochrome system, thereby inhibiting cell metabolism and adenosine triphosphate production. All cells and in particular the liver have a detoxifying process for hydrocyanide with the enzyme rhodenase converting hydrocyanide to thiocyanate, which is then excreted in the urine. This protective system can be overcome by a large amount of cyanide, especially if the patient is also hypovolemic, thereby impairing cyanide metabolism and clearance.^15^,^53^

## SYMPTOMS

Symptoms of carbon monoxide toxicity are usually present when carboxyhemoglobin level exceeds 15%, that is, 15% of the hemoglobin is bound to carbon monoxide rather than oxygen (Table 7). Symptoms are those of decreased tissue oxygenation, with initial manifestations being neurologic.

Major myocardial dysfunction can also develop, especially with preexisting coronary artery disease. In addition, neurologic exposure, by carbon monoxide exposure, can lead to a progressive and permanent cerebral dysfunction. This process is believed to be due to the direct effect of CO on neurons. A brain-demyelinating process results.

More recently, neurons exposed to CO produce nitrous oxide and attract leucocytes, a process suggesting that brain injury is related to inflammation.^54^–^58^ Cyanide toxicity presents in a very similar fashion, with metabolic acidosis and obtundation in severe cases. The degree of toxicity is again dependent on the concentration of HCN in the smoke (Table 8). Diagnosis, however, is more difficult because cyanide levels are not always readily available or reliable.

## DIAGNOSIS

The persistence of a metabolic acidosis in the patient with adequate volume resuscitation and cardiac output suggests the persistent carbon monoxide (or cyanide) impairment of oxygen delivery and utilization. A carboxyhemoglobin level or percent of hemoglobin bound to CO is obtained. However, other cell poisons cannot be excluded. Of importance is that arterial oxygen tension, ***P***_aO_2__, will remain relatively normal because the chemical alteration of hemoglobin by carbon monoxide will not affect the amount of oxygen dissolved in arterial plasma. Therefore, the calculated ***P***_aO_2__ cannot be used as a marker of adequate oxygenation.

Therefore, if there is a discrepancy between the measured ***P***_aO_2__ and measured oxygen saturation, carbon monoxide toxicity is likely present until proved otherwise. However, most times, O_2_ saturation of hemoglobin is calculated, not measured. It is also important to recognize that COHgb looks like HgbO_2_ by color, and values of 5% or greater will lead to a significant overestimation of oxygen saturation using a pulse oximeter.^59^–^60^

A high carboxyhemoglobin also indicates a significant smoke exposure, and therefore, a chemical burn to the airways is likely to be present. A low carboxyhemoglobin does not always indicate a minimal smoke exposure because administration of oxygen at the scene of the fire can displace some of the carbon monoxide before arrival in the emergency department. Blood cyanide levels can be measured to make the diagnosis of cyanide toxicity. Normal levels are less than 0.1 mg/L.

## TREATMENT

**Figure 2:**
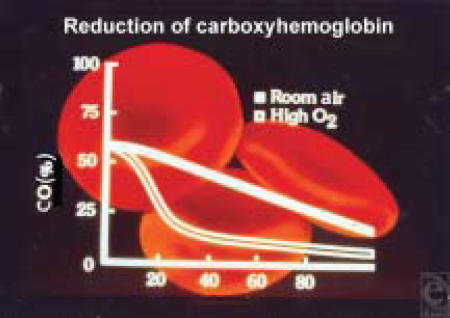
Relationship of COHgb and O_2_ breathed. The half-life of COHgb breathing room air is about 60 minutes, compared with 20 minutes breathing 100% oxygen

Oxygen administration is required in any patient with an index of suspicion of carbon monoxide toxicity. The extra oxygen provided will help displace the carbon monoxide from hemoglobin (Table 9) (Figure 2).

The half-life of COHgb when breathing 100% high-flow oxygen is 20 minutes, that is, the concentration of carboxyhemoglobin is reduced to approximately 50% every 20 minutes.^54^,^56^

Hyperbaric oxygen (2–3 atm) produces an even more rapid displacement and is most useful in cases of prolonged exposure, when it is more difficult to displace carbon monoxide from the cytochrome system.^59^ The drawback of hyperbaric oxygen use is the inability to “get to the burn patient” during the crucial period of hemodynamic and pulmonary instability. Hyperbaric oxygen is best used in cases in which the patient has severe neurologic compromise with high carboxyhemoglobin, more than 50%, but no major burns or severe pulmonary injury, and is not responding to high-flow oxygen with clearance of symptoms. The vast majority of cases can be managed by simply using 100% oxygen. However, there remains a question of the development of a neurologic deficit with a high CO exposure.

Endotracheal intubation and the use of 100% oxygen with mechanical ventilator assistance are indicated for those patients with markedly impaired neurologic function and high carboxyhemoglobin.

Cyanide management remains controversial. In general, cardiopulmonary support is usually sufficient treatment because the liver via the enzyme rhodenase will clear the cyanide from the circulation. Sodium nitrite is used (300 mg intravenously over 5–10 minutes) in severe cases, especially those in which a known diagnosis is made by blood levels. Methemoglobin is produced in this reaction. Methemoglobin does not transport oxygen and some hypoxia can develop. Ordinarily, thiosulfate is also given, which, in turn, binds the cyanide to form thiocyanate. One should be reasonably sure of the diagnosis of cyanide toxicity before giving sodium nitrite.^61^,^62^

Hydroxocobalamin, a vitamin B_12_ derivative found to actively chelate cyanide, is often used in the prehospital management of smoke inhalation victims with a reported considerable improvement in mortality.^61^

### Smoke injury: gas versus particle phase

As described, a number of toxic compounds in smoke are considered to be involved in the lung damage, especially in the airway injury.

Smoke is composed of 2 phases,^6^,^50^,^63^ a gas and a particle phase.

The gas phase contains a number of volatile water-soluble compounds, such as acrolein, chlorine, hydrochloric acid, sulfur compounds, as well as carbon monoxide, carbon dioxide, and cyanide gas.^21^,^42^ Large quantities of short-acting oxidants and oxidant precursors are also present.^31^,^64^ These compounds have been reported to produce proximal airway damage. The gas phase of smoke also contains unusually long-acting oxidants, which can reach distal lung tissues. The particle phase of smoke is composed of carbon particles 0.1 to 10 µm in diameter^31^,^64^–^66^ on which are adhered many of the compounds found in the gas phase along with heavy metals and other oxygen radical-producing compounds and lipid-soluble compounds like the aldehydes. Some of the gas compounds like phosgene can also bind to the carbon.^63^,^67^–^69^ Particle size and tidal volume determine their distribution in proximal versus distal lung.^63^–^70^ Once particles adhere to lung tissue, the injury from adhered toxins, including oxidant release, can occur over hours to days, resulting in progressive cellular injury and in severe injuries, mucus membrane destruction.

A severe airway injury has been noted with wholesmoke exposure, characterized by airway inflammation. Removal of the particles >0.3 µm in diameter nearly eliminates the airways injury,^63^,^69^ indicating that particles of >.3 µm cause the majority of the burn injury. Airway fluid lipid peroxide content is also very high with wholesmoke exposure, suggesting an airway source for the oxidants and resulting oxidant damage.^63^,^68^

An assessment of the importance of the gas versus the particle phase to the smoke injury has only recently been studied as regards protection from the toxins. Numerous studies of fire fighters at the Trade Center explosion have demonstrated chronic lung changes. With nasal breathing, the nasopharynx removes the majority of the particles >5 µm in diameter.^70^ However, with mouth breathing, particle deposition in airways and alveoli, is much greater but again dependent on particle size and tidal volume.^63^,^71^–^74^ A number of investigators have reported increased distal lung and tracheobronchial and distal lung particle deposition with mouth breathing. For particles less than 4 µm in diameter, the greater the volume of breathes, the greater the airway distribution.^71^,^73^ Mouth breathing is likely to occur in a smoke victim because of the nasopharyngeal irritation of smoke, and most smoke injury studies have bypassed the nasopharynx. An unconscious victim also will typically mouth breathe. The degree of singeing of nasal hairs, often seen with smoke exposure, is caused by the local heat from flames and not from the smoke itself. Therefore, the absence of heat injury in no way rules out smoke exposure.

## LUNG DAMAGE FROM SMOKE INHALATION

**Figure 3:**
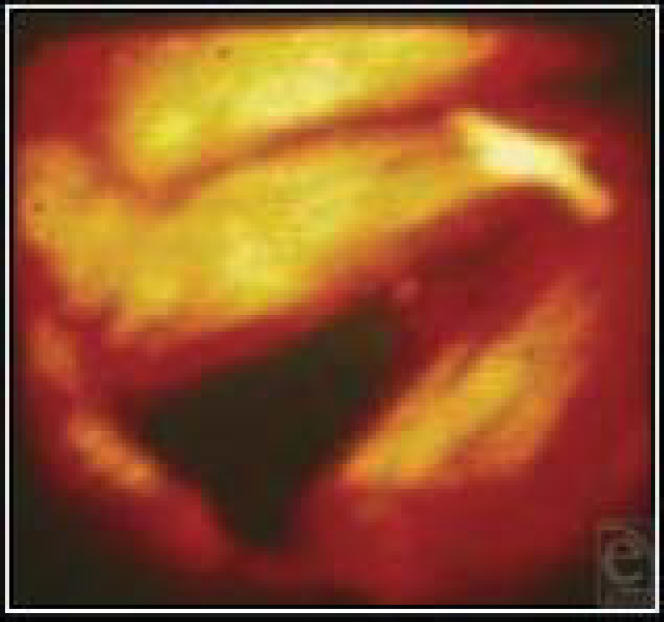
Upper-airways edema after smoke inhalation. Note the erythema and edema of supraglottic tissue and cords. Progression of edema can lead to obstruction

**Figure 4:**
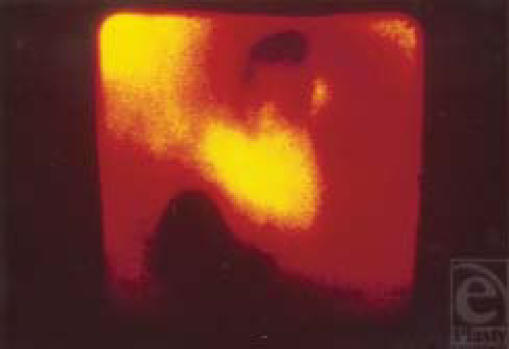
Facial burn (24 hours). Note the marked facial and oropharyngeal distortion caused by the resulting tissue edema

There are indeed 2 major components to our knowledge and treatment of this injury process. The first and most clinically relevant is the physiologic changes that occur and must be managed clinically to obtain optimum outcomes.^75^–^77^ The second component relates to the biochemical changes that are the cause of these physiologic changes.^27^,^28^ Both will be discussed with the first component being a physiologic change following smoke inhalation (Figures 3 and 4).

### Physiologic changes

### Upper airway injury

#### Pathophysiology

Direct-heat injury caused by the inhalation of air heated to a temperature 150°C or higher ordinarily results in burns to the face, oropharynx, and upper airway (above the vocal cords). Even superheated air is rapidly cooled before reaching the lower respiratory tract because of the tremendous heat-exchanging efficiency of the oropharynx and nasopharynx.^78^–^79^

Heat and especially the chemicals in smoke produce an immediate injury to the airway mucosa, resulting edema, erythema, and ulceration. Although these mucosal changes may be anatomically present shortly after the burn, physiologic alteration will not be present until the edema is sufficient to produce clinical evidence of impaired upper-airway patency. This may not occur for 12 to 18 hours. The presence of a body burn magnifies the injury in direct proportion to the size and depth of the skin burn.^80^ The massive fluid requirement necessary to treat the skin burn is partially responsible, as are mediators released from the burned skin. It is now recognized that epithelial cells in the pharynx respond by increasing mucus production, which can then be found in the lower airways often in obstructive mucus clots.

Another compounding injury is any face or neck burn that will produce marked anatomic distortion and, in the case of the deep neck burn, external compression on the larynx. A more superficial burn causes massive external edema but may produce much less mucosal edema and airway compromise. The local edema usually resolves in 4 to 5 days.

The chemical burn to the upper airways results in a spectrum of clinical manifestations during this period. At the very least, a mucosal irritation will persist for several days causing increased cough and mucus production.^81^–^91^

As airway inflammation and bronchial blood flow increased over the several days, even modest volume overload can markedly potentiate the airways edema. The combination of the chemical lung burn and a body burn markedly potentiate the morbidity and mortality of either process. If infection can be controlled and secretions cleared, the acute process will resolve over the next 7 to 10 days. However, the risk of pulmonary infection persists for several weeks, extending well into the inflammation period, as upper airway secretions are aspirated.

The damaged ciliary function of the airways lining leads to an inability to clear secretions and bacteria manifested by a tracheobronchitis.^81^–^91^ Bacterial colonization is inevitable. Characteristically with a severe injury, the damaged mucosa becomes necrotic at 3 to 4 days' injury and slough. The increasingly viscous and copius secretions can lead to increasing distal airway resistance, distal airway obstruction, atelectasis, and a high risk of rapidly developing bronchopneumonia. With modest-to-severe injury, there will be mainly evidence of erythema and edema of the mucosal surface.

#### Symptoms

Symptoms of obstruction, namely, stridor, dyspnea, increased work of breathing, and eventually cyanosis, do not develop until a critical narrowing of the airway is present. Upper airway noise indicative of increased turbulent airway often precedes obstruction. The airway edema and the external burn edema process have a parallel time course so that by the time symptoms of airway edema develop, external and internal anatomic distortion will be extensive.

#### Diagnosis

A history must be obtained regarding the nature of the burn, the presence of smoke, and the patient's initial neurologic status. Inspection of the oropharynx looking for soot or evidence of heat or chemical injury should be done with every burn victim. Direct laryngoscopy is a valuable method to determine whether an injury is present. Typically, erythema and edema will be found. Repeat examinations will be needed if an injury is present and intubation is not performed, because the process often progresses over the next 24 hours. Fiberoptic bronchoscopy is also very useful and can be done very safely.^79^ This diagnostic test is valuable for assessing an airway that is suspect or for evaluating the lung with a known airway burn.

#### Treatment

A very important judgment decision must be made in the initial assessment as to whether the injured airway can be maintained safely without an endotracheal tube. When in doubt of whether progressive edema is likely, it is safest to intubate. These are the major categories of patients at risk for airway compromise.^54^

1. Heat and smoke injury plus extensive face and neck burns:
  This group invariably requires intubation.
2. Oral burn but no smoke injury:
  These patients have difficulty controlling secretions as edema evolves. Early intubation is safe approach because anatomical distortion of the mouth makes intubation at a later period very difficult.
3. Heat and smoke injury, no facial burn:
  If there is no evidence of severe upper-airways edema, this group can be carefully observed. The lack of a facial and mouth distortion makes it feasible to intubate later.

Aerosolized adrenaline has also been found to be beneficial in decreasing the edema process, improving airway patency. It is also beneficial, in both the nonintubated and intubated patients, to maintain a semi-erect position if hemodynamically stable, to minimize the airway and facial edema process. Edema forms much faster than it resolves, so early preventative measures are important.^83^

If intubation is performed, the tube must be well-secured because it may be extremely difficult to replace if it becomes dislodged, as the process of edema evolves. Anticipate a 2- to 3-week period of upper-airway symptomatology as that is the time frame for re-epithelization of the injured mucosa.

## CHEMICAL BURN TO THE LOWER AIRWAYS

### Pathophysiology

This aspect of inhalation injury is often an extension of the upper-airways injury just described but is generally much more serious. Toxins contained in smoke as well as carbon particles coated with irritating aldehydes and organic acids can result in injury to both upper and lower airways. The location of injury will depend on the duration of exposure, the size of the particles, and the solubility of gases. The lungs response to inflammation spills over to systemic organs.

The components in smoke causing injury are defined as follows:

1. The gas phase, as described, contains a host of toxins, including carbon monoxide, cyanide gas, acids, and aldehydes.^84^–^88^ Oxidants are also clearly present in the gas phase.^86^ These agents produce local-airway injury. The vapors, in large part, are mucus membrane irritants leading to intense bronchorrhea, bronchoconstriction, and airway edema. The process often peaks^24^–^36^ hours after injury, although patients with preexisting airway reactivity disease can develop very early intense bronchoconstriction.^21^–^24^
2. The particles phase injury of smoke produces a severe injury. The degree of exposure to the lung is dependent on particle size and breathing pattern.^63^–^66^ The degree of deposition in distal lung is accentuated by deeper breaths, as would be evident in a hypoxic patient or a patient attempting to actively escape the insult. As opposed to the gas phase, which is short-lived, the particulates can adhere to the mucosa and perpetuate the local tissue injury. Particulate clearance, in turn, will be impeded by the impaired mucociliary action perpetuating the injury.^32^–^54^

Smoke from different environments varies dramatically in toxicity. Examples of extremely toxic smoke include smoke from burning automobile interiors, upholstery, and chemical plants in which hydrocarbons are a major component.

### Physiologic changes

**Figure 5:**
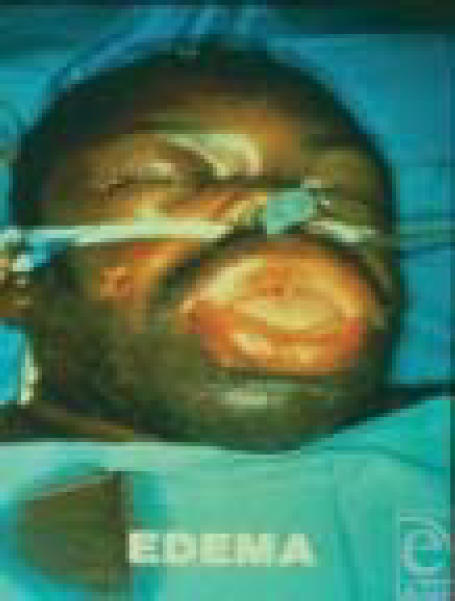
Lower airways response to smoke exposure. Note the presence of erythema and edema in airways encroaching on the airways lumen. Addition of increased mucus can lead to destruction

**Figure 6:**
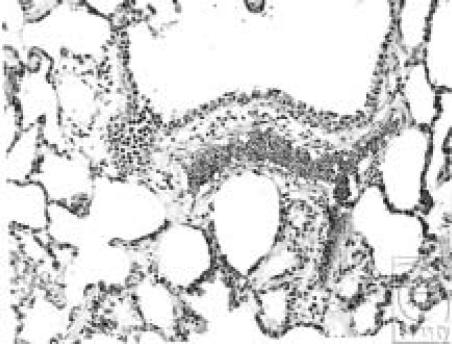
Airway lining at 3 days. Note the infiltration of inflammatory cells around airway

**Figure 7:**
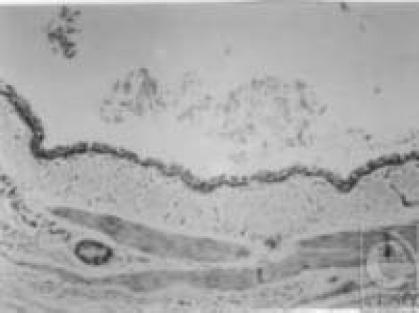
Airway lining at 5 days. Note the absence of airways epithelium and cilia severely impairing immune defenses

There is a marked damage to the airways mucosa. The cell biologic effect will be described later. With moderate-to-severe injuries, there is evidence of denudement of the mucosal surface. Anatomically, erythema and edema are present (Figure 5–7). Histologically, there is evidence of mucosal lining damage and peribronchial inflammation.

### Symptoms

In the first several days after injury, remaining soot continues to be present in the airways secretions. Diffuse rhonchi are usually present, once inflammation develops. Wheezing persists. Continued coughing as well as the residual airways edema and bronchospasms increase the work of breathing, which can lead to fatigue and hypoventilation. Secretions then become tenacious and more difficult to clear. Rales compatible^92^–^95^ with an edema process will be noted in the most severe airways injuries. Evidence of bronchitis is common, followed by bronchopneumonia in a substantial number of patients.

### Diagnosis

Diagnosis of severity of injury is based on the course of the disease process rather than on initial findings from fiberoptic bronchoscopy, which basically indicate only that an injury is present. Chest radiographs during this initial period show, in general, significant underestimation of the severity of lung damage because the injury is usually confined to the airways.^96^ Alveolar injury is seen only in severe cases.

**Figure 8:**
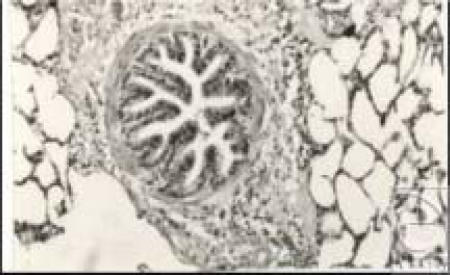
Reactive airways. Note that airways remain hyperactive in the postinhalation injury period. Peribronchial edema and inflammation is evident

**Figure 9:**
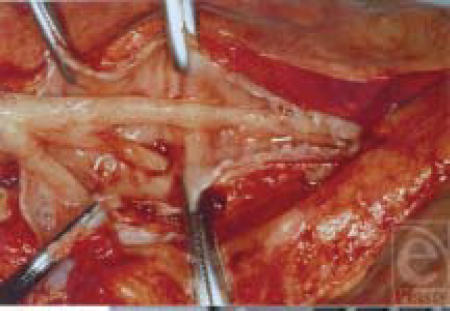
Severe airways injury from smoke. Note the case of airways mucosa, which can break up plugging distal airways

**Figure 10:**
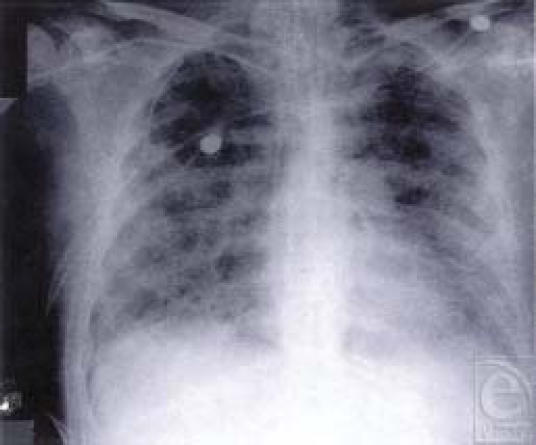
Severe tracheobronchiolitis evolving to bilateral nosocomial pneumonia. Note the diffuse nature of the respiratory dysfunction

Clinical evidence of continued respiratory compromise namely, dyspnea, tachypnea, diffuse wheezing, and rhonchi precede radiographic changes (Figures 8–10). The evidence on radiography of lung damage is usually that of either diffuse atelectasis, pulmonary edema, or bronchopneumonia. Altered gas exchange is reflected in blood-gas analysis, and the assessment of changes in sputum characteristics is a useful parameter to monitor.

### Treatment

The clearance of soot, mucopurulent exudate, mucus plugs, and sloughing mucosa is essential to avoid progression of the lung injury. An endotracheal tube may be necessary if the patient is fatiguing and if gas exchange is worsening. Continued readjustments in tidal volume, rate, and positive end-expiratory pressure are necessary to maintain gas exchange while minimizing barotraumas. Sedation (narcotic-induced or paralysis) may be necessary if the patient's spontaneous ventilatory attempts further impair lung function while on ventilator support. Bronchodilators, by aerosols, are also very helpful, along with frequent changes in position postural drainage. High-frequency percussive ventilation has also been shown to be effective at clearing secretions.^97^ Bronchial lavage has yet to be shown to be an effective method of removing adherent soot particles. Mucolytic agents can be effective if used cautiously to avoid further airways inflammation.

Infection surveillance is crucial during this early period to detect the onset of bacterial bronchitis before the development of pneumonia. Sputum smears monitoring of the character of the sputum are useful early guides. Systemic antibiotics are not given prophylactically but initiated when a bacterial process becomes evident.

In addition, there is a marked increase in airways reactivity resulting in asthma-like symptoms. Airways collapse is much more likely with clots of mucus and neutrophils being released from the upper airways into reactive lower airways.

In severe cases, the airway mucosa separates and pieces plug distal airways leading to a rapidly developing pulmonary dysfunction with increasing airways-resistant and shunt fraction.

## TRACHEOBRONCHIOLITIS AND NOSOCOMIAL PNEUMONIA

### Pathophysiology

The term “nosocomial infection” refers to that which develops in a hospital with no evidence of lung infection present on admission, that is, it is hospital-acquired. Burn patients with a combination of inhalation injury and a major body burn have the greatest risk of pneumonia, with a rate exceeding 50%.^78^–^81^ The high incidence is due to the presence of virulent organisms in the environment and the immunosuppressed state of the burn patient with lung damage. Lung bacterial clearance is significantly impaired in the presence of a burn and inhalation injury. The major impairments in immune dysfunction responsible for lung infections are described.

### Colonization

Nearly 100% of major burn patients with a smoke inhalation respiratory problem have colonized their oropharynx with pathogens. There are a number of routes and events where colonization occurs.^90^–^93^

#### Impaired cough

Impairment of this reflex is a common occurrence. A decrease in the state of consciousness markedly suppresses the need of narcotics for pain control. The impairment is caused by the muscle weakness from catabolism. Any aspirated, infected oral secretions then have the opportunity to proliferate.

#### Impairment of mucociliary action

The airways are lined with ciliated mucus-coated epithelia that beat toward the pharynx, thereby assisting in the continued clearance of particles and microorganisms. This is particularly important to the smaller airways that are less effectively cleared by coughing. The ciliary action is directly injured by heat and chemicals in inhaled smoke.^8^,^32^

#### Airway plugging

The combination of tenacious secretions, mucosal sloughing, and impaired clearance lead to frequent plugging of small- and medium-sized airways causing atelectasis and the increased risk of infection.^98^–^100^

#### Impairment of alveolar macrophage function

Bacteria or particles deposited in the alveoli are rapidly phagocytized by the alveolar macrophage, which destroys them by direct killing via oxygen radical release. A number of factors in the burn patients will impair macrophage function. Inhalation anesthetics, inhalation injury, malnutrition anemia, and hypoxia will impair the macrophage function, thereby increasing the risk of lung infection.

#### Impairment of containment

The postburn immunodeficiency state involves both the cellular and humoral components of resistance, which will impair the ability of the lung defenses to contain infection. Another major factor that impairs the containment process is increased lung water. The movement of edema fluid allows a rapid spread of bacteria to uninvolved areas both as vehicle for carrying bacteria and as an impairment of the sequestration and containment process. Airways and alveolar edema will be present with severe smoke injury.

#### Excess use of antibiotics

The burn patient develops a sepsis syndrome as a result of the inflammatory response to injury. This process makes it difficult to diagnose a superimposed infection often leading to an excessive use of antibiotic and the development of resistant organism.^101^

### Biologic and biochemical changes

#### Upper-airway injury

The pharynx is exposed to both phases of smoke; larger carbon particles, greater than 5 µm in diameter, are deposited in the oropharynx. The key biologic components of the upper-airway tissue damage are described in Table 10.

The upper-airway epithelium has similar barrier functions as the epidermis of the skin. Loss of this barrier will expose the submucosa to smoke particle toxins and local inflammation mediators that can increase the submucosal microvascular permeability. Typically, particles landing in the airway, in the presence of an intact epithelium, are cleared rapidly ending up in the glottis and swallowed. Particles penetrating will need to be cleared by macrophages.^102^

The columnar epithelial begins to denude at about 30 minutes after smoke exposure.^101^–^111^ Toxins that then penetrate will directly increase the local-tissue damage and then the damage occurs from inflammation. Gas-phase toxins are typically cleared on contact with an intact epithelium.

Heat and chemical then increase submucosal vascular permeability.^78^–^83^ This process increases submucosal fluid content^78^–^83^ resulting in edema. It requires about 3 weeks for the damaged epithelium to repair.

A marked increase in tissue inflammation then occurs with the sequestration of a large number of neutrophils, which then will reside in mucus.^106^,^107^ Mediators of inflammation self-perpetuate in the mucosa and submucosa^24^–^29^ although the increased production of airway mucus is an attempt at protection. This upper-airways mucus has been shown to end up in lower airways along with trapped neutrophils leading to thick lower-airway plugs.^81^,^88^

The upper airways vasculature is controlled by adrenergic, cholinergic, and peptidergic nervous mechanism.^85^,^103^–^105^ Sympathetic nerves release norepinephrine and neuropeptide Y, both of which are constrictor agents. Parasympathetic nerves release acetylcholine and usually vasoactive intestinal polypeptide, both of which are vasodilators, the latter being the longer-lasting. These motor nerves are controlled by many reflex inputs. Activation of pulmonary C-fiber receptors by irritants and inflammatory mediators also causes a power vasodilatation. Chemoreceptor reflexes also influence airway vascular tone. Sensory upper nerves in the airway mucosa are responsible for local axon reflexes in response to irritants and inflammatory mediators. These nerves contain neuropeptides such as substance P, neurokinins A and B, and calcitonin gene-related peptide. All these neuropeptides are powerful vasodilators.^103^–^105^

Vasodilation in the presence of an already increased chemical-induced microvascular permeability will markedly accentuate the rate of submucosal edema formation.

#### The bronchial system

With chemical damage, the ciliated epithelium separates from the basement membrane resulting in an instantaneous experiment in immune defenses. In addition, there is a marked increase in bronchial blood flow to the injured airways. Edema rapidly develops in the submucosal space. This edema process occurs in airways as far as the chemical exposure occurs. The edema, in turn, maneuvers the airways lumen resulting in physiologic alteration in airflow. One key component is a marked inflammatory response leading to a large number of white cells that mix with the surface mucosa leading to thick mucus.

There are a number of pathways that are involved with the bronchial edema and bronchoconstriction. One such mechanism is the stimulations of sensory nerves in the airway. A group of agents that are described generally as the neuropeptides are involved.^112^–^114^ The neuropeptides produced in the submucosa after airway injury are potent bronchoconstrictors and can increase blood flow and alter permeability.^38^–^41^ The normal mucosal production of neutral endopeptidases (NEP) is responsible for neutralizing these toxic agents. Loss of NEP by mucosal damage by smoke will lead to an accentuated neuropeptide response.

**Figure 11:**
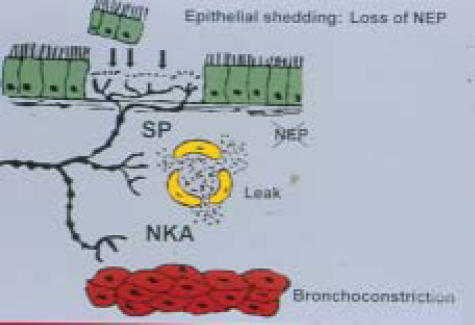
Neuropeptides and airway changes. Note the loss of neutral endopeptidase (NEP) activity due to epithelial damage, increases neuropeptide activity

Restoration of control of potent neuropeptides requires the restoration of columnar epithelium with NEP production, which explains the extensive period of airways hyperactivity after smoke inhalation injury (Figure 11).^112^–^114^ Activation of clotting cascade also leads to increased fibrin production finding its way into the plugs that occlude distal airways. A number of inflammatory cascades also evolve, leading to airway injury. The chemical components in smoke initiate airway inflammation. Prostanoids and leukotrienes are also released especially from mast cells accentuating bronchoconstriction.^115^,^116^

Oxidants or oxygen-free radicals are present in smoke itself and are also produced by chemical reactions in the smoke with oxidants lasting for minutes instead of seconds. Excess oxidants produce a number of responses.^31^,^32^,^45^,^81^–^86^

First oxidants act on the airways mucosa causing cell membrane changes in the lipid component of the membrane. The lipid peroxidation process can be destructive to cell membrane permeability. A release of lipid peroxidation by-products has been shown to be present in lung tissue and lung fluid after smoke exposure. Oxidants will also lead to the activation of inflammation through cytokine release from inflammatory and mast cells, which in turn, leads to further oxidant release and damage.^45^ Local-airway vascular permeability can be increased. The extraordinary high bronchial blood flow with altered permeability can lead to a rapid production of submucosal edema. In addition to oxidant release, there is a marked decrease in antioxidant levels, increasing the risk of oxidant damage.^86^,^81^ A number of antioxidants delivered in an aerosol form have been shown to attenuate the smoke-induced injury.^117^,^118^ Unfortunately, no antioxidant aerosol is currently being used as a standard approach to inhalation injury. Another variable, as mentioned, participating in the process is the 10- to 20-fold increase in bronchial blood flow after smoke exposure. This response results in a large increase in airways edema. In addition, the high blood flow is connected to the pulmonary microvascular blood flow allowing for a rapid distribution of cytokines, oxidants, and so on, from the airways injury to more peripheral parts of the lung including reaching the alveolar capillary membrane.^119^,^120^ Neutrophil adherence to the mucosal layer is a major component. The systemic organ damage, seen with smoke injury, can be in part explained by the systemic circulation carrying proinflammatory mediators via the lung through the bronchial and pulmonary vasculature to systemic organs.^117^

### Alveolar injury

There are a number of vascular changes that occur after smoke injury. Pulmonary vascular resistance is significantly increased by 12 hours after injury, indicating a circulating mediators' response. The increase in venous resistance increases microvascular pressure further accentuating the degree of a capillary leak.^118^,^121^

The increase in neutrophil adherence on the alveolar capillary membrane results in a factory for protease and oxidant release. This response is documented by the presence of the increased proteases and oxidant by products in fluid form from the distal lung.^121^,^122^ There is definitely an increase in alveolar fluid after severe smoke inhalation injury. How much of the fluid is run down from airway bronchorrhea or increased alveolar capillary permeability is difficult to distinguish.

In addition to alveolar flooding, there is also an increase in alveolar collapse as a result of lack of surfactant causing a more rapid alveolar collapse and atelectasis. Atelectasis is a common component of smoke injury.^123^

### Treatment

At the present time, treatment for the biologic and biochemical changes that occur in the alveolus remains respiratory supports against the deleterious physiologic changes. A number of antioxidants and anti-inflammatory blockers have been shown to decrease mucosal and alveolar edema and atelectasis. These include a variety of antioxidant aerosols, cytokine inhibitors, and neutrophil-adherence inhibitors.^118^–^121^

Improvement of pulmonary pathology has been demonstrated with this variety of approaches. Currently, these approaches have not been adopted as clinical modulators but remain mainly as research tools to study the disease process.

## SYSTEMIC RESPONSE TO SMOKE INHALATION

There are well-described systemic responses to a smoke inhalation injury. These responses are most pronounced in the presence of a body burn where burn mortality rate is markedly increased in the presence of a smoke inhalation injury. A moderate smoke inhalation injury has been reported to decrease initial systemic oxygen delivery and oxygen consumption mainly by elevated carboxyhemoglobin levels, as well as a recognized decrease in cardiac functions.^124^,^125^ However, this response is followed by an increase in systemic oxygen consumption, when COHgb is removed, as a hypermetabolic state occurs in response to the lung injury. During this time period, there is a selective decrease in blood flow to the intestine and pancreas potentially leading to future organ failure especially from bacterial translocation. The increased blood flow likely occurs to soft tissues and muscle. The process of selective changes in blood flow is likely driven by inflammatory mediators.^126^ In addition, systemic fluid requirements are increased as also the evidence of a transient change in systemic vascular permeability in soft tissues, which is similar to a septic response.^127^,^128^ This effect is not due to carbon monoxide or cyanide but is likely the systemic response to mediators from the lung inflammatory process.

An increase in oxidant stress, as measured by markers of the lipid peroxidation process, is noted in liver, kidney, and blood levels in several studies.^129^–^134^ In addition, tissue antioxidant activity is decreased likely due to increased consumption by the increased oxidant activity. The systemic effect appears quite transient, lasting 24 to 48 hours in the absence of a burn.

The major cause of death today from burn injury is respiratory failure. Pneumonia is a common complication. However, there are significant early changes in physiology. First, studies have shown that the combined injury leads to a greater than 50% increase in systemic oxygen consumption compared with a burn alone.^125^,^128^,^135^ Second, early fluid requirements are increased over 100% in the initial resuscitation period suggesting that there is a further change in vascular permeability in both burn and nonburn tissue from the lung injury. In addition, burn edema is markedly increased with the presence of smoke inhalation. The systemic changes are caused by the particle phase of smoke because the removal of particles with smoke exposure to just the gas phase prevents the accentuated burn response. The degree of increased burn-tissue edema is dependent on the degree of airways injury, the greater the smoke insult, the greater the burn edema.^127^ Carbon monoxide and cyanide do not appear to play a role.

Interestingly, oxygen consumption has been reported to be oxygen-delivery dependent with the combination injury as opposed to a burn alone, increasing the development of tissue hypoxia. The inability to extract more oxygen from hemoglobin, when needed, is a typical response to sepsis, ARDS, or any generalized inflammatory process again indicating the close relationship between systemic and lung physiology in the presence of lung inflammation.^129^ These responses are clearly inflammation-induced, from the smoke injury process.

As previously described, oxygen radicals are involved in the systemic response.^132^–^136^ Smoke-induced radicals can be long-lived either providing the opportunity to produce direct systemic effects or an alternate explanation would be the release of systemic proinflammatory cytokines that would then produce systemic inflammation.

Of interest is the presence of a marked increase of by-products of lipid peroxidation in systemic tissues, caused by oxidant damage to all membranes in burn tissue edema fluid from the combined injury compared with burn alone. A similar process is seen in the lung with the combined injury.^132^,^136^

One hypothesis, backed up by research data, is that this increased overall oxidant activity can be attenuated. Deferoxamine starch, an iron chelator given in resuscitation fluid attenuates the systemic response to free iron which activates oxygen radical production to smoke injury. The same protection is provided when deferoxamine starch is delivered to the smoke-exposed lung by aerosol.^137^ These findings would indicate that the lung itself is the source of the free iron. Interestingly deferoxamine, a small molecule provided alone, has no effect indicating that a large starch complex is needed, which likely has a much longer half-life on the airways mucosa.

Of importance is the fact that maneuvers that are, as previously described, used to decrease the smoke-induced lung injury will significantly attenuate the deleterious systemic effects. At present, however, it is not possible to accurately distinguish whether the increased morbidity and mortality of the combined injury is due primarily to the lung injury inducing severe systemic changes or vice versa. However, evidence, as described, would favor the hypothesis that the inflammatory process in the lung, caused by smoke exposure, is the driving force in the response.

## SUMMARY

Smoke inhalation injury is a complex multifaceted lung and systemic disease process, which until recently, has been poorly understood. It was recognized as a major killer of burn victims in the early 1900s. But improved respiratory support modalities developed in the 1960s were needed before the prolonged course of injury was appreciated, namely, the delayed mucosal and alveolar damage. With ARDS being defined, the lung as an inflammatory organ began to be appreciated.

The toxins in smoke began to be better defined through the 1970s. Of particular importance was the recognition that these chemicals, including oxidants, attached to smoke particles where, after being deposited in the large and small airways, they initiated an injury process. Finding particles in the lungs through bronchoscopy became a marker for a delayed progressive process. Carbon monoxide and cyanide are carried in the gas phase to the alveolus, where they are absorbed through the alveolar capillary membrane.

The physiologic response to smoke exposure was further identified in the past 2 decades. Small airway obstruction, by mucus and inflammatory cell casts, is quite characteristic whereas sloughing of mucosa is evident in more severe cases. Increased airways reactivity for prolonged time periods is characteristic. Increased work of breathing alveolar flooding or collapse with resulting impaired gas exchange is also characteristic, as is increased pulmonary infection.

More recent advances have focused on the lungs' inflammatory reaction to the smoke insult. A host of mediators are involved including proinflammatory cytokines, neuropeptides, activated clotting factors, and very importantly, oxidant release. Oxidants are components of smoke itself, produced by reactions in smoke and again produced by the inflammatory process. New advances in the understanding of the biochemical changes present in the lung responsible for injury has yet to evolve into new treatment modalities, reflecting the typical lag time between science and clinical practice.

Several antioxidant aerosols have been shown to be experimentally effective. Aerosol development seems to be a logical way to improve therapy, which focuses on physiologic support. Finally, the recognition that a smoke inhalation injury is also a systematic injury process adds valuable information on management and better answers the question as to why the mortality rate for the combined smoke and burn injury is so much greater, than either one alone.

With our ever-increasing understanding of the biology of this injury, we become closer to developing treatment modalities focused on lung inflammation in addition to current physiologic support measures.

## Figures and Tables

**Figure 1:**
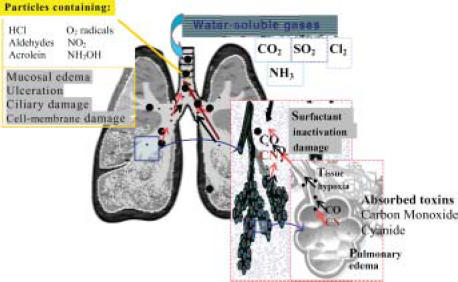
Effect of the components of smoke on the lungs. Water-soluble gases are seen producing upper-airway irritation. The components on the carbon particles lead to more severe airways damage including cell membrane changes and, in some cases, alveolar damage. Carbon monoxide and cyanide are absorbed directly into the blood from the alveoli

**Table 1:** Notable events leading to smoke inhalation knowledge

Event	Characteristics
World War I	Use of poisonous gases and the effect on the lungs
Cleveland Clinic Fire, 1929	Effect of the inhalation of volatile products from burning x-ray film
Coconut Grove Fire, 1942	Effect of volatile products in smoke causing early and late respiratory distress, initially from upper airway compromise and then airway plugging
Mid-1940s World War II	Pathophysiologic; time, course, and treatment using respiratory assistance
1950s–1960s	Development of blood–gas monitoring and intensive care
Vietnam War, 1960s	Identification of adult respiratory distress syndrome caused by alveolar capillary membrane damage
1970s	Better understanding of smoke inhalation causing post-traumatic pulmonary insufficiency (PTPI, ARDS), improved ventilator management, and the toxicology of smoke
1980s–1990s casualties from hotel fire in Las Vegas and Kings Cross Underground Station Fire	Role of the effect of airway inflammation in smoke inhalation injury
2000s mass casualties, World Trade Center Disaster 2001	Long-term effects of smoke exposure changes in airways epithelium
Rhode Island Nightclub Fire, 2003	Biochemical and cell biologic changes; improved ventilatory strategies

**Table 2:** Historic progression of known physiologic changes in smoke injury

• Volatile products in smoke leading to early and late respiratory distress
• Airways edema leading to early destruction and later airways plugging
• Use of blood gases to assess the exchange of gases and critical care to stabilize lung physiology
• Ventilatory support to stabilize physiologic changes
• Concept of tracheobronchitis and alveolitis impairing ventilation and gas exchange
• Adult respiratory distress syndrome, alveolar edema and collapse, surfactant deficiency, increase in shunting
• Role of airways inflammation and mediators on the physiologic changes with smoke inhalation

**Table 3:** Common components of smoke and their effect

Products in smoke	Effect
Carbon dioxide	Increased respiratory drive
Carbon monoxide	Tissue hypoxia, organ failure, death
Hydrogen cyanide	Tissue hypoxia, organ failure, death
Oxygen radicals	Mucus membrane damage, alveolar damage
Acrolein or propenal	Irritant to necrosing agent, involving airways mucosa death
Aldehydes, formaldehyde, acetaldehyde, butyraldehyde	Necrosing agent to mucosa, denatures protein
Ammonia	Mucus membrane irritant, including airway muscosa
Sulfur dioxide	Mucus membrane irritant
Hydrogen chloride (phosgene)	Necrosing airway mucosa
Aromatic hydrocarbons, eg, benzene	Mucus membrane irritant, systemic toxin
Hydrogen sulfide	Mucus membrane irritant and corrosive

**Table 4:** Origin of selected toxic compounds

Material	Source	Decomposition products
All combustible products		Carbon monoxide, dioxide, oxygen radicals
Cellulose	Wood, paper, cotton	Aldehydes, acrolein
Wool, silk	Clothing, fabric, blankets, furniture	Hydrogen cyanide, ammonia, hydrogen sulfide
Rubber	Tires	Sulfur dioxide, hydrogen sulfide, oxygen radicals
Polyvinyl chloride	Upholstery, wire/pipe coating, wall, floor, furniture coverings	Hydrogen chloride, phosgene
Polyurethane	Insulation, upholstery material	Hydrogen cyanide, isocyanates, ammonia, acrylonitriles
Polyester	Clothing, fabric	Hydrogen chloride
Polypropylene	Upholstery, carpeting	Acrolein, oxygen radicals
Polyacrylonitrile	Appliances, engineering, plastics	Hydrogen cyanide
Polyamide	Carpeting, clothing	Hydrogen cyanide, ammonia
Polyamine resins	Household and kitchen goods	Hydrogen cyanide, ammonia, formaldehyde
Acrylics	Aircraft windows, textiles, wall coverings	Acrolein, aldehydes
Fire retardants	Polymeric materials	Hydrogen cyanide, acetylene chloroethane, propene nitrite

**Table 5:** Effects of inhalation of hydrogen chloride on humans

Hydrogen chloride concentration in air, ppm	Symptoms
1–5	Limit of odor
5–10	Mild irritation of mucus membranes
35	Irritation of throat on short exposure
50–100	Barely tolerable
1000	Lung edema after short exposure

**Table 6:** Relationship of CO in smoke to percent COHgb

CO concentration, ppm	Smoke characteristics	Time to 20% COHgb
10,000	Heavy smoke	<5 min
5,000	Moderate	<10 min
2,000	Mild smoke	20 min

**Table 7:** Carbon monoxide intoxication

Carboxyhemoglobin level, %	Symptoms
0–5	Normal value
15–20	Headache, confusion
20–40	Disorientation, fatigue, nausea, visual changes
40–60	Hallucination, combativeness, coma, shock state
>60	Mortality > 50%

**Table 8:** Relation of hydrogen cyanide concentrations in air and symptoms in humans

HCN concentration, ppm	Symptoms
0.2–5.0	Threshold of odor
10	Maximum safe exposure
18–36	Slight symptoms (headache)
45–54	Tolerated for ½–1 h
100	Fatal – 1h
110–135	Fatal in ½–1 h
180	Fatal in > 10 min
280	Immediately fatal

**Table 9:** Treatment of carbon monoxide and cyanide toxicity

Carbon monoxide – awake	Carbon monoxide – obtunded	Cyanide
High flow by mask oxygen (FiO_2_ 100%) until carboxyhemoglobin < 10%	Intubate	Cardiovascular support
	100% oxygen via positive pressure ventilation	Sodium nitrite only if not responding and high likelihood of diagnosis HCN toxicity
	Hyperbaria used if patient not responding to 100% (specific indications remain unclear)	Sodium thiosulfate

**Table 10:** Biologic changes in upper airway with smoke injury^102^–^111^

• Destruction of epithelial layer
• Increased vascular permeability
• Increased edema formation
• Increased neuron stimulation
• Increased mucus production
• Tissue inflammation

## References

[1] Traber D, Pollard V, Herndon D (2002). Pathophysiology of Inhalation Injury. Total Burn Care.

[2] Sachor F, Amllory G (1963). Lung lesions in patients dying of burns. Arch Pathol.

[3] Thompson P, Herndon D (1986). Effect on mortality of inhalation injury. Trauma.

[4] Winternatz MC (1920). Pathology of War Gas Poisoning.

[5] Wollstein M, Meltzer S (1918). Experiment chemical pneumonia. J Exp Med.

[6] Nichols B (1930). The clinical affects of the inhalation of nitrogen dioxide. AJR.

[7] Cope D (1943). Care of the victims of the Coconut Grove fire at the Massachusetts General Hospital. N Engl J Med.

[8] Saffle J (1993). The 1942 fire at Boston's Coconut Grove Nightclub. Am Surg.

[9] Harkins HN (1942). The treatment of burns.

[10] Moore F, Lyons J, Pierce E (1969). Post-Traumatic Pulmonary Insufficiency: Pathophysiology of Respiratory Failure and Principles of Respiratory Care After Surgical Operations, Trauma, Hemorrhage, Burns and Shock.

[11] Zukria B, Ferre J, Floch N (1972). The chemical factors contributing to pulmonary damage in smoke poisoning. Surgery.

[12] Fine J, Frank ED, Ravin H (1959). The bacterial factor in traumatic shock. N Eng J Med.

[13] Kointz A, Allen M (1929). On the relationship of bacteria to so-called chemical pneumonia. J Exp Med.

[14] Ashbaugh D, Bigelow D, Petty T, Levine B (1967). Acute respiratory failure in adults. Lancet.

[15] Symington I (1978). Cyanide exposure in fires. Lancet.

[16] Zukira B, Weston C, Chodoff M (1972). Smoke and carbon monoxide poisoning in fire victims. J Trauma.

[17] Herndon D, Thompson P, Traber D (1985). Pulmonary injury in burned patients. Crit Care Clin.

[18] Thompson P, Herndon D, Traber D, Abton S (1986). Effect on the mortality of inhalation injury. J Trauma.

[19] Navar P, Saffle J, Warden G (1985). Effect of inhalation injury on fluid resuscitation requirements after thermal injury. Am J Surg.

[20] Moylan J, Chan CK (1977). Inhalation injury: an increasing problem. Ann Surg.

[21] Kinsella J, Carter R, Reid WH (1991). Increased airway reactivity after smoke inhalation. Lancet.

[22] Micak R, Cortielia J, Desai M, Herndon D (1997). Lung compliance, airway resistance and work of breathing in children after inhalation injury. J Burn Care Rehabil.

[23] Prezant D, Weidon M, Banach G (2002). Cough and bronchial responsiveness in firefighters at the World Trade Center site. New Engl J Med.

[24] Park G, Park J, Jeong Jeong S (2003). Prolonged airway and systemic inflammatory reactions after smoke inhalation. Chest.

[25] Finnerty C, Herndon D, Przkora R (2006). Cytokine expression profile over time in severely burned pediatric patients. Shock.

[26] Traber D, Maybauer D, Herndon D (2005). Inhalational and acute lung injury. Shock.

[27] Maybauer M, Maybauer D, Fraser J (2006). Recombinant human activated protein Cimproves pulmonary function in ovine acute lung injury resulting from smoke inhalation and sepsis. Crit Care Med.

[28] Syrkina O, Quinn D, Jang W (2007). Inhalation of JNK activation prolongs survival after smoke inhalation from fires. Am J Physiol.

[29] Harrangston D, Biff L, Cigffi W (2005). The station nightclub fire. J Burn Care Rehabil.

[30] Pryor W (1992). Biological effects of cigarette smoke, wood smoke and the smoke from plastics. Free Radic Biol Med.

[31] Einhor I (1976). Physiological and toxicological aspects of smoke produced during the combustion of polymeric materials. Environ Health Perspect.

[32] Hoffman N, Oettel N (1969). Comparative toxicology of thermal decomposition products. Mod Plastics.

[33] Prien T, Traber D (1988). Toxic smoke compounds and inhalation injury, a review. Burns Incl Thermal Inj.

[34] Lee W, Mayberry K, Crapo R, Jensen R (2000). The accuracy of pulse oximetry in the emergency department. Am J Emerg Med.

[35] Dirk C, Brown D, Donaldson K, Stone V (2003). The role of fire radicals in the toxic and inflammatory effects of four different ultrafine particle types. Inhal Toxicol.

[36] Raub J, Mathew-Nolf M, Hampson N, Thom S (2000). Carbon monoxide poisoning – a public health perspective. Toxicology.

[37] Myers R, Linberg S, Cowley R (1979). Carbon monoxide poisoning: the injury and its treatment. J ACEP.

[38] Vogel S, Sultran T (1981). Cyanide poisoning. Clin Toxicol.

[39] Jones J, McMullen J, Daugherty J (1987). Toxic smoke inhalation: cyanide poisoning in fire victims. Am J Emerg Med.

[40] Lundquist P, Dammer L, Sorbo B (1989). The role of hydrogen cyanide and carbon monoxide in fire casualties: a prospective study. Forensic Sci Int.

[41] Terrell J, Montgomery R, Reinhart C (1978). Toxic gases from fires. Science.

[42] Wald P, Balmes J (1987). Respiratory effects of short-term, high-intensity toxic inhalations: smoke, gases, and fumes. J. Intensive Care Med.

[43] Chu C (1989). New concepts of pulmonary burn injury. J Trauma.

[44] Takeuchi K, Kato M, Suzuki H (2001). Acrolein induces activation of the epidermal growth factor receptor of human keratinocytes for cell death. J Cell Biochem.

[45] Pryer W, Biological effects of cigarette smoke, wood smoke, and the smoke from plastics: the use of electron spin resonance (1992). Free Radic Biol Med.

[46] Lachorki T, Church D, Pryor W (1988). Persistent free radicals in the smoke of common household materials; biological and clinical implications. Environ Res.

[47] Austin C, Wang D, Ecobrichon D, Dussault G (2001). Characterization of volatile organic compounds in smoke at experimental fires. J Toxicol Environ Health A.

[48] Shimagu T (1988). A dose response model of smoke inhalation injury. Ann. Surg.

[49] Schwartz DA (1987). Acute inhalation injury. State art Rev Occup Med.

[50] Parkes W (1982). Occupational Lung Disorders.

[51] Gold A, Burges W (1978). Exposure of firefighter to toxic air contaminants. Am Ind Hyg Assoc J.

[52] Bruce M, Burqe E (2006). Analysis of factors that influence rates of carbon monoxide uptake, distribution and washout from blood and extravascular tissue using a multicompartmental model. J Appl Physiol.

[53] Alarie Y (2002). Toxicity of fire in smoke. Crit Rev Toxicol.

[54] Prockop L, Chichkova R (2007). Carbon monoxide intoxication: an updated review. J Neurol Sci.

[55] Haponik E (1992). Smoke inhalation injury: some priorities for respiratory care professionals. Resp Care.

[56] Kim J, Chang K, Song I (2003). Delayed encephalopathy of acute carbon monoxide intoxication: diffusivity of cerebral white matter lesions. Am J Neuroradiol.

[57] Gorman D, Drewry A, Huang YL, Sames C (2003). The chemical toxicology of carbon monoxide. Toxicology.

[58] Durak AC, Coskun A, Yikilmaz A (2005). Magnetic resonance imaging findings in chronic carbon monoxide intoxication. Acta Radiol.

[59] Johnson A (2005). Hyperbaric oxygen for carbon monoxide poisoning: a systematic review and critical analysis of the evidence. Toxicol Rev.

[60] Bozeman W, Myers R, Barish R (1998). Confirmation of the pulse oximetry gap in carbon monoxide poisoning. Ann Emerg Med.

[61] Fortin JL, Giocanti JP, Ruttimann M, Kowalski JJ (2006). Prehospital administration of hydroxocobalamin for smoke inhalation-associated cyanide poisoning. Clin Toxicol (Phila).

[62] Baud F, Barricot P, Toffis B (1991). Elevated blood cyanide concentrations in victims of smoke inhalation. N Eng J Med.

[63] Lalonde C, Demling R, Brain J, Blanchard J (1994). Smoke inhalation injury in sheep is caused by the particle phase not the gas phase. J Appl. Physiol.

[64] Lalonde C, Picard L, Youn YK, Demling Rh (1995). Increased early postburn fluid requirements and oxygen demands are predictive of the degree of airways injury by smoke inhalation. J Trauma.

[65] Haponik EF, Summer WR (1987). Respiratory complications in burned patients: pathogenesis and spectrum of inhalation injury. J. Crit Care.

[66] Kaufman JW, Scherer PW, Yang CC (1996). Predicted combustion product deposition in the human airway. Toxicology.

[67] Hill IR (1996). Reactions to particles in smoke. Toxicology.

[68] Lalonde C, Picard J, Campell C, Demling R (1994). Lung and systemic oxidant and antioxidant activity after graded smoke exposure in the rat. Shock.

[69] Hales CA, Musto SW, Janssens S (1992). Smoke aldehyde component influences pulmonary edema. J Appl Physiol.

[70] Gamsu G, RM Weintraub, Nadel JA (1973). Clearance of tantanium from airways of different caliber in man evaluated by a roentgenographic method. Am Rev Respir Dis.

[71] Demling RH, Blanchard J, Lalonde C (1994). Effect of increasing tidal volume of smoke breaths on smoke-induced lung dysfunction. J Appl Physiol.

[72] Hales CA, P Barkin, Jung W (1989). Bronchial artery ligation modifies pulmonary edema after exposure to smoke with acrolein. J Appl. Physiol.

[73] Demling RH (1993). Effect of graded increases in smoke inhalation injury in the early systemic response to a body burn. Crit Care Med.

[74] Foord, N, Black A, Walsh M (1978). Regional deposition of 2.5–7.5 µm diameter inhaled particles in healthy male non-smokers. J Aerosol Sci.

[75] Sallsten G, Gustafson O, Johnson L (1992). Experimental wood smoke inhalation: particle associated changes in alveolar macrophages. Toxicol Pathol.

[76] Moores H, Janigan D, Hajeela R (1992). Lung injury after experimental smoke inhalation: particle associated changes in alveolar macrophages. Toxicol Pathol.

[77] Hantson P, Btera R, Clemessy J (1997). Early complications and value of initial clinical and paraclinical observations in victims without burns. Chest.

[78] Clark W, Bonaventura M, Meyers W (1989). Smoke inhalation and airway management at a regional burn unit. J Burn Care Rehabil.

[79] Moylan J, Alexander L (1978). Diagnosis and treatment of inhalation injury. World J Surg.

[80] Navar P, Saffle J, Warden G (1985). Effect of inhalation injury on fluid resusciatation requirements after thermal injury. Am J Surg.

[81] Rouby J (1992). Nasocomial bronchopneumonia in the critically ill. Am Rev Resp Dis.

[82] Pereira W, Kovnat DM, Snider GL (1978). A prospective cooperative study of complications following flexible fiberoptic bronchoscopy. Chest.

[83] Ziegler D, Bent G (2001). Caustic induced upper airway obstruction responsiveness to nebulized adrenaline. Pediatrics.

[84] Sexton I, Pronchik D (1998). Chlorine inhalation: the big picture. J Toxicol Clin Toxicol.

[85] McMullen M, Hetrick T, Cannon L, Haddad L (1998). Ammonia, nitrogen, nitrogen oxides and related compounds. Clinical Management of Poisoning and Drug Overdose.

[86] Youn Y, LaLonde C, Demling R (1992). Oxidants and the pathophysiology of burn and smoke inhalation injury. Free Radic Biol Med.

[87] LaLonde C, Ulhas N, Demling R (1997). Plasma catalase and glutathione levels are decreased in response to inhalation injury. J Burn Care Rehabil.

[88] Demling R, LaLonde E (1993). Relationship of body burn induced lipid peroxidation on the degree of injury after smoke inhalation and a body burn. Crit Care Med.

[89] Mitchelson BP (1992). The electron energy-loss spectroscopic analysis of inhaled smoke particles. J Microsc.

[90] Ibriham E (2000). A comparative analysis of patients with early onset versus late onset nosocomial pneumonia in the ICU setting. Chest.

[91] Demling R, Wolfort S, Meakins J (1994). Early post-operative pneumonia. Surgical Infections.

[92] Stenton S, Kelly C, Walters E, Hendrick D (1988). Induction of bronchial hyperresponsiveness following smoke inhalation injury. Br J Dis Chest.

[93] Livingston D (2002). Prevention of ventilator associated pneumonia. Am J Surg.

[94] Murakami K, Taber D (2003). Pathophysiological basis of smoke inhalation injury. News Physiol Sci.

[95] Hollingsed T, Saffle J, Briton R (1993). Etiology and consequences of respiratory failure in thermally injured patients. Am J Surg.

[96] Lee M (1988). The plain chest radiograph after acute smoke inhalation. Clin Radiol.

[97] Hall J, Hunt J, Arnolds B, Purdue G (2007). Use of high frequency persuasive ventilation in inhalation injuries. J Burn Care Res.

[98] Micak R, Herndon D, Herndon D (2002). Respiratory care. Total Burn Care.

[99] Oldenberg F, Dolovich M, Montgomery J (1979). Effects of postural drainage, exercise and cough on mucous clearance in chronic bronchitis. Am Rev Resp Dis.

[100] Palmeiri T, Jackson W, Greenhalgh D (2002). Benefits of early tracheostomy in severe burned children. Crit Care Med.

[101] Fabian T (2000). Empiric therapy for pneumonia in the surgical intensive care unit. Am J Surg.

[102] Luppman M, Yeatis D, Albert R (1980). Deposition, retention and clearance of inhaled particles. Br J Ind Med.

[103] Tari C, Baranink J (2002). Upper airway neurological mechanisms. Curr. Opin Allergy Clin Immunol.

[104] Widderombre J (1991). Neural control of airway vasculation and edema. Am Rev Respir Dis.

[105] Widderombre J (1990). The NANC system and airway vasculation. Arch Int Pharmacodyn Ther.

[106] Cox R, Micak R, Chanke D (2007). Upper airway mucus deposition in lung tissue of burn trauma victims. Shock.

[107] Cox R, Barke A, Katusaka S (2003). Airway destruction in sheep with burn and smoke inhalation injuries. Am J Respir Cell Mol Biol.

[108] Abdi S, Evans M, Cox R (1990). Inhalation injury to tracheal epithelium in an ovine model of cotton smoke exposure. Early phase 20 min. Am Rev Resp Dis.

[109] Wong C, Evans M, Cox R (1992). Morphologic changes in basal cells during repair of tracheal epithelium. Am J Pathol.

[110] Barrow R, Wang C, Yang S (1994). Efficacy of cefaxolin in promoting ovine tracheal epithelial repair. Respiration.

[111] Barrow R, Wang C, Evans M, Herndon D (1993). Growth factors accelerate epithelial repair in sheep trachea. Lung.

[112] Barnes P, Baraniuk J, Belvise M (1991). Neruopeptides in the respiratory tract. Am Rev Resp Dis.

[113] Traber L, Herndon D (1990). Peptide mediation of the bronchial blow flow elevation following inhalation injury. Care Shock.

[114] Lundberg S, Martling C (1983). Cigarette smoking, induced airways edema due to activation of capsaicin sensitive vagal afferents and substance P release. Neuroscience.

[115] Jin LJ, LaLonde C, Demling RH (1986). Lung dysfunction after thermal injury in relation to prostanoid and oxygen radial release. J Appl Physiol.

[116] Janssens S, Musto S, Hutchinson W (1994). Cycloxygenase and lipoxygenase inhibition by BW-775C reduces acrolein smoke-induced acute lung injury. J Appl Physiol.

[117] Stothert J, Ashley K, Kramer G (1990). Intrapulmonary distribution of bronchial blood flow after moderate smoke inhalation. J Appl Physiol.

[118] LaLonde C, Ikegami K, Demling R (1994). Aerosolized deferoxamine prevents lung and systemic injury caused by smoke inhalation. J Appl Physiol.

[119] Efimova O, Volokhov A, Iliaifar S, Hales C (2000). Ligation of the bronchial artery in sheep attenuates early pulmonary changes following exposure to smoke. J Appl Physiol.

[120] Sakuri H, Johnigan R, Kikuchi Y (1998). Effect of reduced bronchial circulation on lung fluid flux after smoke inhalation in sheep. J Appl Physiol.

[121] Katahira J, Murakami K, Schmalstieg FC (2002). Role of anti-L-selectin antibody in burn and smoke inhalation injury in sheep. Am J Physiol Lung cell Mol Physiol.

[122] Alpard SK, Zwischenberger JB, Tao W (2000). New clinically relevant sheep model of severe respiratory failure secondary to combined smoke inhalation/cutaneous flame burn injury. Crit Care Med.

[123] Nieman G, Clark W, Wax S (1980). The effect of smoke inhalation on pulmonary surfactant. Ann Surg.

[124] Abde S, Traber P, Herndon D (1995). Effects of ibuprofen on airway vascular response to cotton smoke injury. Eur J Pharmacol.

[125] Demling R, LaLonde C (1990). Moderate smoke inhalation produces decreased oxygen delivery, increased oxygen demands, and systemic but not lung parenchymal lipid peroxidation. Surgery.

[126] Sakuri H, Traber P (1998). Altered systemic organ blood flow after combined injury with burn and smoke inhalation. Shock.

[127] Demling R, LaLonde C, Youn YK, Picard L (1995). Effect of graded increases in smoke inhalation injury in the early systemic response to body burn. Crit Care Med.

[128] LaLond C, Picard L, Youn Y, Demling R (1995). Increased early postburn fluid requirements and oxygen demands are predictive of the degree or airways injury by smoke inhalation. J Trauma.

[129] Demling R, Knox J, Youn Y, LaLond C (1992). Oxygen consumption early postburn becomes oxygen delivery systemic response to a burn body. J Trauma.

[130] Traber D, Hawkins H, Enkhbaatar P (2007). The role of the bronchial circulation in the acute lung injury resulting from burn and smoke inhalation. Pulm Pharmacol Ther.

[131] Cox R, Burke A, Traber D, Herndon D, Hawkins H (2007). Production of pro-inflammatory polypeptides by airway mucous glands and its potential significance. Pulm Pharmacol Ther.

[132] Demling R, LaLonde C, Picard L, Blanchard J (1994). Changes in lung and systemic oxidant and antioxyidant activity after smoke inhalation. Shock.

[133] LaLonde C, Picard L, Campbell C, Demling R (1994). Lung and systemic oxidant and antioxidantactivity after graded smoke exposure in the rat. Circ shock.

[134] Demling R, Ikegami K, LaLonde C (1995). Increased lipid peroxidation and decreased antioxyidant activity correspond with death after smoke exposure in the rat. J Burn Care Rehab.

[135] LaLonde C, Nayak U, Hennigan J, Demling R (1997). Plasma catalase and gluathione levels are decreased in response to inhalation injury. J Burn Care Rehabil.

[136] LaLonde C, Knox J, Youn YK, Demling R (1992). Burn edema is accentuated by a moderate smoke inhalation injury in sheep. Surgery.

[137] Demling R, Picard L, Campbell C, LaLonde C (1993). Relationship burn-induced lung lipid peroxidation on the degree of injury after smoke inhalation and body burn. Crit Care Med.

